# Assay Strategies for the Discovery and Validation of Therapeutics Targeting *Brugia pahangi* Hsp90

**DOI:** 10.1371/journal.pntd.0000714

**Published:** 2010-06-15

**Authors:** Tony Taldone, Victoria Gillan, Weilin Sun, Anna Rodina, Pallav Patel, Kirsty Maitland, Kerry O'Neill, Gabriela Chiosis, Eileen Devaney

**Affiliations:** 1 Department of Molecular Pharmacology and Chemistry, Sloan Kettering Institute, New York, New York, United States of America; 2 Parasitology Group, Division of Veterinary Infection and Immunity, Institute of Comparative Medicine, University of Glasgow, Glasgow, United Kingdom; 3 Department of Pharmaceutical Sciences, College of Pharmacy and Allied Health Professions, St. John's University, Jamaica, New York, New York, United States of America; Biomedical Research Institute, United States of America

## Abstract

The chemotherapy of lymphatic filariasis relies upon drugs such as diethylcarbamazine and ivermectin that largely target the microfilarial stages of the parasite, necessitating continued treatment over the long reproductive life span of the adult worm. The identification of compounds that target adult worms has been a long-term goal of WHO. Here we describe a fluorescence polarization assay for the identification of compounds that target Hsp90 in adult filarial worms. The assay was originally developed to identify inhibitors of Hsp90 in tumor cells, and relies upon the ability of small molecules to inhibit the binding of fluorescently labelled geldanamycin to Hsp90. We demonstrate that the assay works well with soluble extracts of *Brugia*, while extracts of the free-living nematode *C. elegans* fail to bind the probe, in agreement with data from other experiments. The assay was validated using known inhibitors of Hsp90 that compete with geldanamycin for binding to Hsp90, including members of the synthetic purine-scaffold series of compounds. The efficacy of some of these compounds against adult worms was confirmed *in vitro*. Moreover, the assay is sufficiently sensitive to differentiate between binding of purine-scaffold compounds to human and *Brugia* Hsp90. The assay is suitable for high-throughput screening and provides the first example of a format with the potential to identify novel inhibitors of Hsp90 in filarial worms and in other parasitic species where Hsp90 may be a target.

## Author Summary

Helminth diseases of humans remain a major problem in many parts of the tropics. Treatment of these parasitic infections is restricted to a limited number of drugs and few new compounds are in development. One of the major obstacles to the development of new therapeutics is the lack of high-throughput screens that can be adapted to parasitic species for the identification of small molecule inhibitors. Here we present a simple, inexpensive assay for the identification of inhibitors of Hsp90 in filarial worms. The assay, first described for the identification of Hsp90 inhibitors in tumor cells, does not require recombinant protein but relies upon the ability of a fluorescently labelled drug to bind to Hsp90 in the context of a soluble fraction of worm homogenate. We validated the assay using known inhibitors of Hsp90, including derivatives of the synthetic purine-scaffold series of Hsp90 inhibitors and were able to show a differential sensitivity to these compounds between human and *Brugia* Hsp90.

## Introduction

Lymphatic filariasis (LF) caused by the nematode parasites *Wuchereria bancrofti* and *Brugia malayi* remains a major tropical disease with an estimated 120 M individuals infected [1]. The infection is transmitted to humans by the bite of a mosquito carrying infective third stage larvae (L3) in the head and mouthparts. The L3 enter the lymphatics and develop through two moults to sexually mature adults; following mating, the adult female worm produces an abundance of first stage larvae (L1 or microfilariae, Mf) which circulate in the bloodstream and which represent the reservoir of infection for the mosquito host. There are no vaccines available for preventing infection. The control of LF is not easy and relies upon drugs that largely target the Mf, such as diethylcarbamazine (DEC), a drug developed in 1947 [2], or ivermectin. This necessitates continued treatment over the long reproductive life span of the worm, as Mf re-populate the blood stream from adult worms that are largely unaffected by these drugs. The development of a macrofilaricidal compound has long been a goal of the World Health Organization (WHO), but attempts to develop appropriate compounds have yet to be successful [3]. Meanwhile the ongoing campaign for the global elimination of LF is based on the use of DEC, or ivermectin in sub-Saharan Africa where LF overlaps with onchocerciasis, together with albendazole, a drug with known efficacy against gastro-intestinal nematodes but with limited efficacy against filariae [4]. The availability of a macrofilaricidal drug would obviate the need for continued treatment with microfilaricidal drugs. As well as the financial implications of long-term drug delivery programmes, repeated exposure to chemotherapy poses credible risks for the development of resistance, as is apparent from the reduced efficacy of ivermectin in some onchocerciasis patients [5].

Despite the fact that DEC and more recently ivermectin have been extensively used to treat LF, their precise mode of action remains unclear. In fact there is a dearth of information on appropriate drug targets for the chemotherapy of LF, and while the mode of action of ivermectin on the free-living model nematode *Caenorhabditis elegans* is well-documented [6], [7] its target in parasitic nematodes is still open to debate [8], [9]. The only novel chemotherapeutic target in filarial nematodes currently under development is the *Wolbachia* endosymbiont [10], [11]. However, the availability of the *B. malayi* genome sequence [12] may facilitate the identification of novel drug targets [13]. The dearth of drugs available to treat LF, and indeed other helminth infections of humans [1] reflects a number of limitations: the lack of availability of high-throughput screening (HTS) systems, our limited knowledge of how existing drugs kill filarial worms, and the paucity of investment in these specific areas.

We have previously identified the molecular chaperone Hsp90 as a possible target in LF [14]. Hsp90 is a validated drug target in many cancers [15], and an emerging target in neurodegenerative diseases [16] and in viral and fungal infections [17]. It has an unusual ATP-binding pocket in the N-terminal domain, and several small molecules that bind competitively with ATP in this site inhibit its function [18]. With inhibitor bound, the conformation adopted by Hsp90 destabilises client proteins, which are then targeted for degradation via the proteasome pathway [15]. As many clients of Hsp90 are essential proteins within cells of pathogenic function, inhibition of the Hsp90 pathway is invariably lethal to these, but not to normal cells [15].

Exposure of *Brugia pahangi* to geldanamycin (GA), a specific inhibitor of Hsp90 [18], kills adult worms and Mf *in vitro*. Interestingly, this agent has no significant effect on the related nematode *Caenorhabditis elegans* [19], despite a high degree of conservation between the two Hsp90 sequences [20]. The low affinity of *C. elegans* Hsp90 for GA is supported by studies in yeast, as an *hsp90* null strain complemented with *C. elegans hsp90* is relatively resistant to GA [21]. In contrast, expression of *C. elegans* Hsp90 in mammalian cells does not confer GA-resistance on the cells, as the nematode Hsp90 forms hetero-dimers with the endogenous (GA sensitive) Hsp90, thus retaining sensitivity to GA [19]. While puzzling in light of the high structural similarity between Hsp90 from *C. elegans* and *B. pahangi*, recent studies on Hsp90 in human cancer and normal cells bring some molecular insights into this paradox. Namely, it is becoming evident that a complex array of co-chaperones and post-translational modifications modulate Hsp90 activity distinctly in each cellular context, and these factors dictate the sensitivity of Hsp90 to small molecule inhibitors such as GA [22].

While our previous findings position Hsp90 as a potential target in killing both adults and Mf of *B. pahangi*, the use of GA as a drug in treating these infections is prohibited by several unfavorable chemical features. First, it exhibits severe hepatotoxicity, which has been associated with the benzoquinone ring and imposes strict dosing limitations. Secondly, it is metabolically and chemically unstable. Also, it has very low solubility in aqueous media resulting in formulations requiring DMSO [18]. In cancers, much effort has been directed at discovering novel Hsp90-inhibitors of distinct chemical scaffold, and currently several such agents are in pre-clinical or clinical evaluation (reviewed in [23]). Similar efforts are required for the development of agents targeted at *Brugia* Hsp90. Since the sensitivity to Hsp90 inhibitors is determined by a complex crosstalk between Hsp90 and the cellular environment, it is necessary to develop screening strategies that account for the endogenous presentation of Hsp90 in *Brugia*.

In this paper we describe an assay amenable for such a screening strategy for inhibitors of filarial worm Hsp90. The assay relies on fluorescence polarization (FP) and upon the ability of inhibitors to block the binding of fluorescently labelled GA to Hsp90 species characteristic of *Brugia* homogenates. We show that the assay is specific and sensitive and able to detect compounds that bind in the *Brugia* Hsp90 ATP pocket. The assay is suitable for high-throughput screening, does not require the production of purified recombinant protein and could be adapted to screen Hsp90 inhibitors against other parasites, where Hsp90 is known to be a valid target [24], [25].

## Methods

### Reagents

Cy3B-GA was synthesized as previously reported [26], [27] and was dissolved in DMSO to form a 10 µM solution. GA, radicicol, ivermectin and novobiocin were purchased from Sigma. The assay buffer (HFB) contained 20 mM HEPES (K), pH 7.3, 50 mM KCl, 5 mM MgCl_2_, 20 mM Na_2_MoO_4_, and 0.01% NP40. Before each use, 0.1 mg/mL bovine gamma globulin (Panvera Corporation, Madison, WI) and 2 mM DTT (Fisher Biotech, Fair Lawn, NJ) were freshly added. The synthesis and characterization of PU-scaffold Hsp90 inhibitors is presented elsewhere [27], [28].

### Preparation of worm extracts

Adult worms of *B. pahangi* were obtained from infected gerbils, exactly as described previously [14] while *C. elegans* was cultured on agar plates at 20°C [29]. Adult worms of *B. pahangi* or mixed stage *C. elegans* worms were frozen in liquid nitrogen, ground in a pestle and mortar to a fine powder and re-suspended in an appropriate volume of HFB assay buffer. The protein concentration was estimated using the BioRad protein assay. At this point lysates were freeze-dried for shipping to the USA. In some experiments *C. elegans hsp90* was knocked down by RNAi using the standard bacterial feeding protocol [30], exactly as described previously [20]. Control plates were seeded with HT115 cells transformed with empty double T7 vector, L4440, while RNAi plates were seeded with HT115 cells containing L4440 expressing a 294 bp fragment of *C. elegans daf-21* (*hsp90*). *C. elegans* L4 worms were added to the plates and incubated at 20°C for 24 h, following which worms were washed off the plates and processed for the FP assay as described above. The efficiency of *(hsp90)RNAi* was assessed by immunoblotting relative to the actin signal exactly as described previously [31].

### Human cancer cell lysate preparation

The human breast cancer cell line SKBr3 was obtained from the American Type Culture Collection (Manassas, VA). Cells were cultured in DME HG:F-12 medium supplemented with 10% fetal bovine serum, NEAA, 1% penicillin, and streptomycin. Cells were collected and frozen to rupture the membranes and then dissolved in binding buffer with added protease and phosphotase inhibitors to form the lysate. Lysates were stored at –80°C before use. Total protein content was determined using the bicinchoninic acid assay kit (Pierce Biotechnology, Rockford, IL) according to the manufacturer's instructions.

### FP measurements

FP measurements were performed using black 96-well microtiter plates (Corning #3650), where both the excitation and the emission occurred from the top of the well, on an Analyst GT plate reader (Molecular Devices, Sunnyvale, CA). An integration time of 100 ms was used, and Z height was set at 3 mm (middle). The excitation polarization was set at static, and the emission polarization was set at dynamic. For cy3B-GA, an excitation filter at 530 nm and an emission filter at 580 nm were used with a dichroic mirror of 561 nm. All FP values were expressed in millipolarization (mP) units. The mP values were calculated using the equation mP  = 1000× [(*I*S – *IS*B) – (*I*P – *I*PB)]/[(*I*S – *IS*B) + (*I*P – *I*PB)], where *I*S is the parallel emission intensity measurement, *I*P is the perpendicular emission intensity sample measurement, and *I*SB and *I*SP are the corresponding measurements for background (buffer). Total fluorescence was determined as 2×*I*P+*I*S.

### Assay development and optimization

The Hsp90 FP assay was carried out in black 96-well microplates in a total volume of 100 µL in each well. To determine the equilibrium binding of cy3B-GA and worm lysates, increasing amounts of lysates were incubated with 6 nM of cy3B-GA. The plate was incubated at 4°C for different time periods, and the FP in mP was measured with the Analyst GT.

To test the assay stability, the binding experiment was measured after different incubation times at 4°C for up to 24 h. The assay window was calculated as the difference between the FP value recorded for the bound cy3B-GA and the FP value recorded for the free cy3B-GA (defined as mP – mP_f_). Specific binding was defined as the contribution of bound ligand to signal, and was calculated as b × mP_b_  =  mP – f × mP_f_, where b and f are the fractions of bound and free tracer, respectively; mP is the recorded polarization value for a particular Hsp90 concentration; and mP_f_ is the polarization value for free tracer.

For assay performance, the signal-to-noise ratio (S:N) and the Z' factor were calculated based on the following equations: S:N  =  (μ_b_ – μ_f_)/(SD_b_^2^ + SD_f_^2^)^0.5^, where SD_b_ and SD_f_ are the standard deviations for bound (b) (lysate + cy3B-GA) and free (f) cy3B-GA (cy3B-GA only), respectively; μ_b_ – μ_f_ is the difference in mean signals for bound and free cy3B-GA; and Z' factor  = 1 – (3SD_b_ + 3SD_f_)/(μ_b_ – μ_f_), where the Z' factor reflects the quality of the assay itself without the intervention of test compounds [32].

### Competition FP assays

For the competition studies, FP assays were performed as previously reported [26]. Briefly, the Hsp90 inhibitors dissolved in DMSO were added at several concentrations to the HFB assay buffer containing both 6 nM cy3B-GA and nematode lysate (2 µg *B. pahangi* lysate) or human cancer cell lysate (3 µg SKBr3 lysate) in a final volume of 100 µL. Drugs were added to triplicate wells. Free cy3B-GA (6 nM cy3B-GA), bound cy3B-GA (6 nM cy3B-GA + lysate, as indicated above) and buffer only containing wells (background) were included as controls in each plate. Plates were incubated on a shaker at 4°C, and polarization values measured at 8 to 24 h. Percentage inhibition was calculated as follows: (% Control)  = 100 – ((mP_c_ – mP_f_)/(mP_b_ – mP_f_))×100, where mP_c_ is the recorded mP from compound wells, mP_f_ is the average recorded mP from cy3B-GA–only wells, and mP_b_ is the average recorded mP from wells containing both cy3B-GA and lysate, and plotted against values of competitor concentrations. The inhibitor concentration at which 50% of bound cy3B-GA was displaced was obtained by fitting the data using a nonlinear regression analysis as implemented in Prism 4.0 (Graphpad Software).

### Data analysis

All experimental data were analyzed using SOFTmax Pro 4.3.1 and plotted and analyzed using Prism 4.0 (Graphpad Software Inc., San Diego, CA).

### Effect of inhibitors on *B. pahangi* viability

Two inhibitors belonging to the purine-scaffold group of Hsp90 inhibitors, PU-H71 and PU-DZ8, were tested for their effects on adult and microfilarial stages of *B. pahangi* exactly as described previously [14]. Briefly, six adult female worms of *B. pahangi* were cultured individually in 24-well plates in RPMI-1640 with 10% heat inactivated foetal calf serum containing drug, carrier alone at the appropriate concentration (DMSO) or as a positive control, GA at 1.0 µM. Compounds PU-H71 and PU-DZ8 were tested at the indicated concentrations. Mf output by individual female worms was assessed at two time points and adult worms were examined microscopically and their condition noted. Results are expressed as mean Mf output over a three-day period ± SD. Statistical significance between groups was calculated using the Mann Whitney test with P values <0.05 being considered significant.

## Results

### *Brugia pahangi* assay development

To measure the affinity of cy3B labelled GA to Hsp90 species characteristic of *Brugia*, we took advantage of an FP assay developed for tumor cell Hsp90 [33]. FP is based on the observation that when a relatively small, fast-tumbling fluorescent labeled compound (in this case a fluorescently labeled GA molecule) is excited with plane-polarized light, the emitted light is random with respect to the plane of polarization, resulting in a lower mP value. When the compound is bound to a bigger molecule (in this case Hsp90), the complex tumbles more slowly and the emitted light is polarized, resulting in a higher mP value. Thus, the change in mP reflects the interaction between the labeled compound (i.e. fluorescent GA) and the protein (i.e. Hsp90). The mP value is proportional to the fraction of bound ligand and the assay is very powerful in measuring real-time protein–inhibitor interactions in solution.

**Figure 1:**
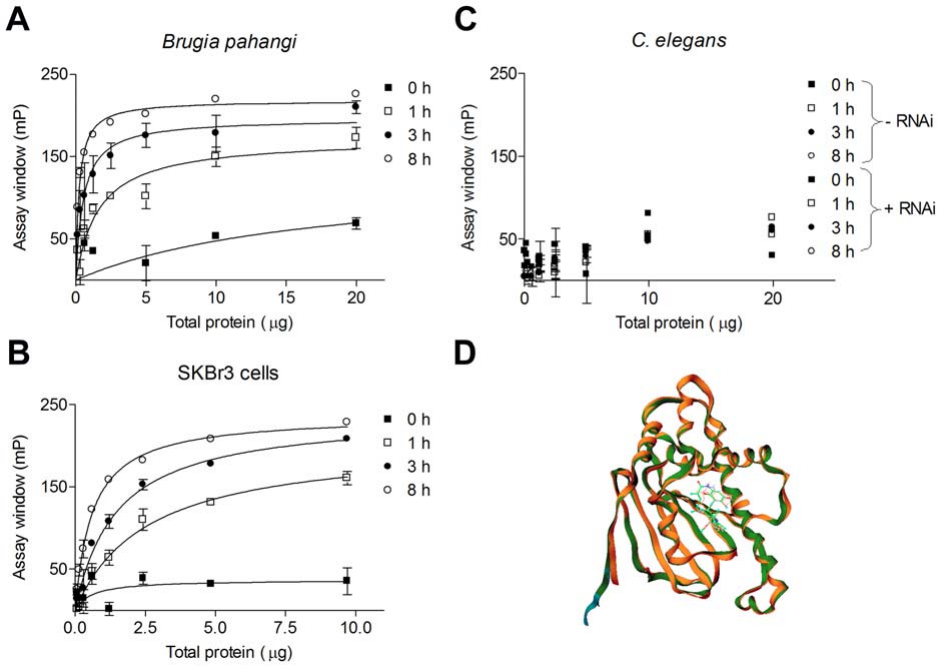
*Brugia* assay development. (**A–C**) Dose-response curve for the binding of 6 nM cy3B-GA to Hsp90 present in the adult *Brugia pahangi* worm extract (A), SKBr3 cell lysate (B) and *C. elegans* extract (-RNAi) or to a *C. elegans* extract in which *hsp90* had been depleted by RNAi by approximately 40% (+RNAi) (C) Various amounts of total lysate protein dissolved in binding buffer (0–20 µg/well) were incubated in triplicate wells with the ligand at 4°C, and the response was measured at the indicated time intervals. Fluorescence polarization was read with an Analyst GT instrument. Values obtained at several time intervals were plotted against the amount of added total protein. The assay window data were obtained by subtracting free tracer values from values recorded in the presence of specified protein concentrations. Data were analyzed and plotted in Prism 4.0. Points, mean; bars, s.d. (**D**) Overlay of GA-bound homology models (derived using Prime software of Schrodinger L.L.C, NY) of *B. pahangi* (orange, Accession number AJ005784) and *C. elegans* (green, Accession number Z75530) and the X-ray crystal structure of human Hsp90α (blue, PDB ID: 1YET).

Here we investigated whether the FP assay could be applied to a more complex, multicellular species such as a nematode worm. To investigate this possibility, we prepared extracts from *B. pahangi* and from the GA-insensitive nematode *C. elegans*, and probed the affinity of the fluorescent GA molecule, cy3B-GA, for the Hsp90 species characteristic of these two nematodes (Figure 1). For an FP assay to be useful, the binding affinity of the fluorescent ligand to the protein should be high and the binding range or the assay window (maximum mP at saturation - minimal mP at no protein) should be large (>100 mP). Rewardingly, cy3B-GA bound to the *Brugia* Hsp90 species (Figure 1A) with a high affinity, similar to the tumor Hsp90 species (Figure 1B). At reduced lysate concentrations, a low mP value was obtained for both extracts; as the concentration of lysate increased, a greater fraction of fluorescent GA bound to Hsp90 species and polarization progressively increased to reach saturation. The signal amplified with time, reached equilibrium at 8 h and remained stable thereafter, resulting in an excellent assay window of approximately 200 mP (Figure 1A). In contrast, cy3B-GA bound the *C. elegans* extracts with very low affinity (Figure 1C). The signal at each time point was virtually indistinguishable from time 0, indicating that the low increase in mP, observed especially at high extract concentrations, was caused by non-specific binding of the tracer or by autofluorescence from the worm extract. This conclusion was further supported by the fact that *C. elegans* extracts from worms where Hsp90 levels were reduced by ∼40% by means of RNAi (data not shown), showed essentially no change in binding signal (Figure 1C). These assay results concord well with previous observations that despite a high degree of conservation between the two nematode Hsp90 sequences [20] and an essentially overlapping binding mode predicted computationally (Figure 1D), GA has no significant effect on *C. elegans* [19].

**Figure 2:**
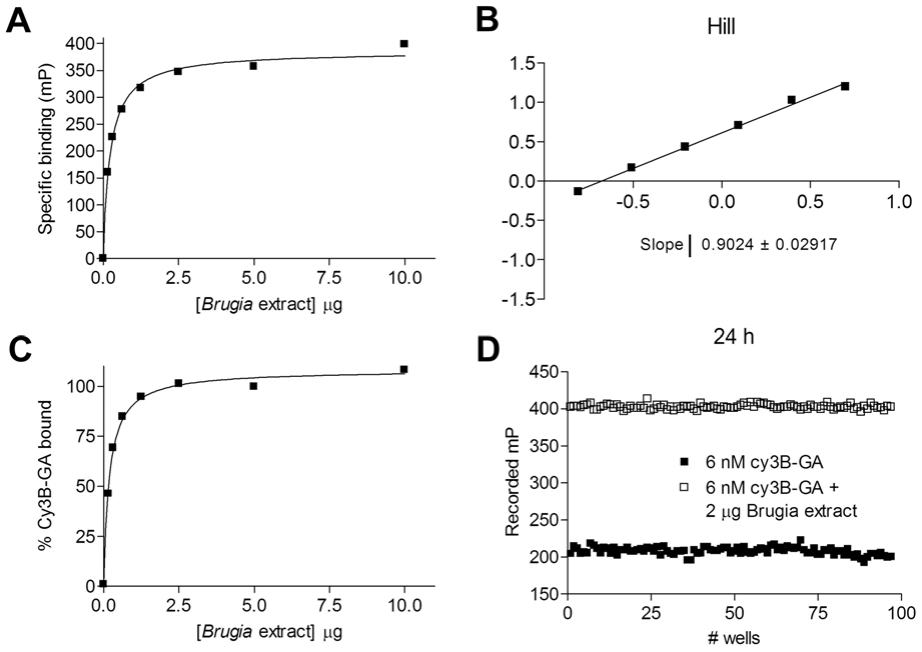
Analysis of *Brugia* assay performance. (**A–C**) Data collected at equilibrium in the binding experiment described in Fig1A were transformed and analyzed using a nonlinear regression method in Prism 4.0, and Hill plots were constructed. Specific binding represents the contribution of bound ligand to total recorded values. (**D**) Two 96-well plates each containing 48 free tracer control wells (6 nM cy3B-GA) and 48 bound tracer control wells (6 nM cy3B–GA with added lysate, 2 µg/well) were used to determine the suitability of the assay for high-throughput screening. The millipolarization value for each well was recorded, and average values corresponding to each plate were plotted. The signal-to-noise ratios and the Z' factors were calculated as indicated in Methods.

Analysis of the binding curve at equilibrium, as demonstrated by specific binding (Figure 2A) and Scatchard and Hill plot analyses (Figure 2B and data not shown) demonstrated that at the low lysate amounts required to reach saturation, the interaction from other cellular material was precluded. Even at 1.5–2.5 µg *Brugia* extract, more than 95% to 99% of cy3B-GA was Hsp90-bound (Figure 2C).

To evaluate the applicability of the 96-well FP assay for HTS, the assay performance parameters S:N and Z' were determined to be 32 and 0.87, respectively (Figure 2D). For FP, a S:N value higher than 8 is recommended for reliable readings. The Z' factor is a parameter for the quality of the assay itself without test compounds. Assays with a Z' factor between 0.5 and 1.0 are considered to be reliable, robust, and suitable for HTS [32].

### *Brugia pahangi* assay validation

**Figure 3:**
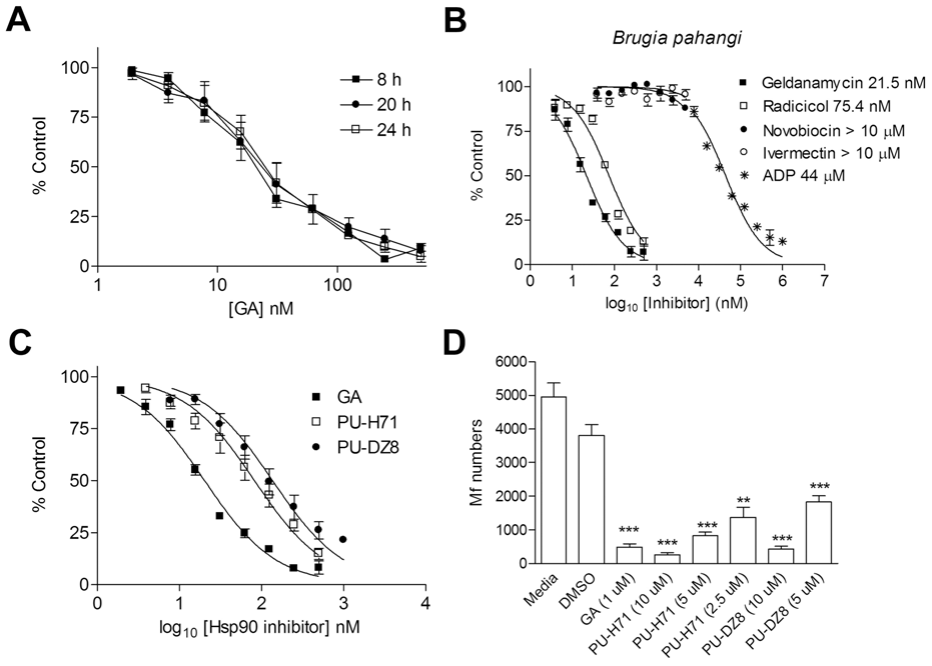
*Brugia* assay validation. (**A–C**) Increasing concentrations of indicated inhibitors were added in triplicate to the reaction buffer containing 6 nM of cy3B-GA and *Brugia* extracts (2 µg/well) in a final volume of 100 µL. Free (6 nM cy3B-GA) and bound (6 nM cy3B-GA with 2 µg/well *Brugia* extract) controls were included on each plate. The polarization values were measured after incubation at 4°C for the indicated times to evaluate assay stability (**A**) or for 24 h with the indicated inhibitors to evaluate their affinity for *Brugia* Hsp90 (**B, C**). The competitive effect was expressed as percentage of control and was calculated by dividing the millipolarization (mP; subtracting free cy3B-GA) value from inhibitor wells by the average mP (subtracting free cy3B-GA) from controls (cy3B-GA and cell lysate with vehicle DMSO) in each plate. Ligand binding was plotted against the log_10_ inhibitor concentration, and EC_50_ values were calculated using a nonlinear least-square curve-fitting program in Prism 4.0. Points, mean; bars, s.d. (**D**) Six adult female *B. pahangi* were incubated individually in 2.0 ml of tissue culture medium containing GA at 1.0 µM, PU-H71 at 10, 5 or 2.5 µM, PU-DZ8 at 10 or 5 µM, DMSO or medium alone. Graphs show mean and SD of Mf output over a three-day period from six female worms per group. Data combined from two separate experiments. *** P<0.005 for all drug concentrations vs DMSO except for PU-H71 at 2.5 µM where P = 0.0260 (**).

To further demonstrate the specificity of the recorded signal for Hsp90, we conducted several competitive assays (Figure 3). As expected, unlabelled GA displaced cy3B-GA binding to *Brugia* extracts in a dose-dependent manner, resulting in near 100% displacement at 250–500 nM (Figure 3A). The competitive assay signal remained constant over the recorded interval of 8 to 24 h (Figure 3A). Only competitive binders of the N-terminal binding site of Hsp90, such as GA, radicicol and the endogenous ligand ADP, resulted in dose-dependent reduction of the FP signal (Figure 3B). In contrast, the Hsp90 C-terminal interactor, novobiocin, or the anthelmintic ivermectin had no such effect. Next we evaluated two derivatives of the purine-scaffold class, a chemotype distinct from that of the natural products GA and radicicol [34]. PU-H71 and PU-DZ8, representative of the purine-scaffold compounds, are fully synthetic molecules that were rationally designed to bind to the N-terminal site ATP pocket of Hsp90. Both agents inhibited the binding of cy3B-GA to *Brugia* Hsp90, albeit with a potency 4- to 8-fold lower than GA, respectively (IC_50_^PU-H71^  = 80 nM and IC_50_^PU-DZ8^  = 160 nM vs IC_50_^GA^  = 20 nM) (Figure 3C).

To investigate whether the results of the FP assay correlate with the effects of Hsp90 inhibitors on *Brugia* viability and Mf output, the effect of two PU-scaffold compounds on worm viability was assessed. Adult female worms of *B. pahangi* were incubated in medium containing either GA at 1 µM [14], or the two synthetic inhibitors PU-H71 and PU-DZ8 at 5 and 10 µM. All agents had a profound effect on Mf release (Figure 3D), with a trend in potency that followed their affinity for *Brugia* Hsp90 (GA > PU-H71 > PU-DZ8 see Figure 3C). Adult worms were markedly less active in 10 µM and 5.0 µM PU-H71 and 10 µM PU-DZ8, but there was less of an effect at 5.0 µM PU-DZ8. In the wells that were affected, the adult worms become elongate and immobilised, very similar to the reported effects of GA on adult *Brugia*. This experiment was repeated on two additional occasions with similar results. In a further experiment, adult worms were exposed to the most potent of the purine-scaffold compounds, PU-H71, at 5.0 and 2.5 µM and to GA at 1.0 µM and Mf production assessed. The most significant reductions in Mf output were observed at 1.0 µM GA and at 5.0 µM PU-H71. At 2.5 µM PU-H71 the reduction in Mf output, while still significant, was less pronounced (see Figure 3D). In separate experiments, Mf were purified from the peritoneal cavity of infected animals and incubated alone in each of the drugs. At 10 µM PU-H71, 100% of worms were dead by day 7 and at 5.0 µM PU-H71, approximately 80% of worms were dead by day 7. For 10 µM PU-DZ8 at day 7, ∼95% of worms were dead with 50% mortality at 5.0 µM. In the control wells there was ∼5% mortality at these times, while Mf incubated with GA (1.0 µM) were all dead.

**Figure 4:**
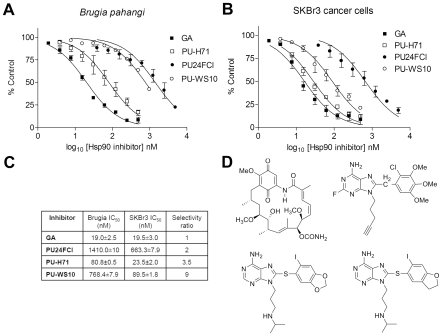
*Brugia* assay identifies species selective Hsp90 inhibitors. (**A–C**) Increasing concentrations of indicated inhibitors were added in triplicate to the reaction buffer containing 6 nM of cy3B-GA and *Brugia* extracts (2 µg/well) (A) or SKBr3 cell lysates (3 µg/well) (B) in a final volume of 100 µL. Free (6 nM cy3B-GA) and bound (6 nM cy3B-GA with 2 µg/well *Brugia* or 3 µg/well SKBr3 extract) controls were included on each plate. The polarization values were measured after incubation at 4°C for 24 h with the indicated inhibitors to evaluate their *Brugia* and human tumor Hsp90 affinity. Points, mean; bars, s.d. EC_50_ values were determined as shown in Fig. 3, and tabulated to indicate the selectivity ratio for the two Hsp90 species (C).

To evaluate whether the assay is sufficiently sensitive to differentiate between human and *Brugia* Hsp90 species, and thus lead to the identification of species-selective inhibitors, we measured the affinity of GA and of several closely related PU-class derivatives, PU-24FCl, PU-H71 and PU-WS10 for the *Brugia* (Figure 4A) and the SKBr3 (Figure 4B) Hsp90 complexes. While GA interacted with identical affinity with both human and *Brugia* species, a good selectivity was noted in the PU-series, with a selectivity ratio of 2, 3.5 and 9 for PU-24FCl, PU-H71 and PU-WS10, respectively (Figure 4C). The 3-fold change in selectivity ratio between PU-H71 and PU-WS10, two agents of almost identical chemical structure, further confirms the sensitivity of the Hsp90 pocket to the cellular environment and suggests that it may be possible to identify molecules that specifically target the parasite Hsp90.

## Discussion

Although high-throughput screening is now well established for the identification of small molecules with novel activities in many human diseases, relatively little attention has been paid to the application of such methods to the neglected tropical diseases of humans [35], [36]. Consequently the development of novel therapeutics for most of these diseases has lagged behind other areas. In an attempt to redress that balance, several academic institutes and public-private partnerships have developed screening programmes which are beginning to bear fruit [37]. Notable recent successes are the identification of the oxadiazoles for the control of schistosomiasis [38] the development of kinase inhibitors for the chemotherapy of malaria [39], and the identification of a range of targets for *Trypanosoma brucei* (reviewed in [40]). A recent paper also demonstrated promising results using an existing chemical library to screen against a novel target in filarial worms. Here it was shown that the veterinary anthelmintic, closantel, had activity against a chitinase from the filarial nematode, *Onchocerca volvulus* [41]. In this paper we describe the application of an assay developed for the identification of inhibitors of tumor cell Hsp90 to the parasitic nematode *Brugia*. Through its simple mix-and-read format, and the use of low amounts of fluorophore and cell homogenates, the FP assay is low-cost and thus highly amenable for high-throughput screening. Cy3B is a red-shifted dye that has been found to be particularly well suited for FP due to its increased fluorescence intensity, fluorescence lifetime of 2.9 ns, and stability of its signal in a variety of aqueous solvent conditions. In addition, its use in the labeling of GA may limit the potential interference of fluorescent small-molecule library components and reduce false-positives resulting from light scattering caused by insoluble compounds.

Instead of recombinant protein, the original tumor cell Hsp90 assay made use of human cancer cell lysates, but nonetheless, could selectively probe the interaction of small molecules with cancer cell-specific Hsp90 by taking advantage of the specificity of GA for tumor Hsp90 species. While its use is now validated in several tumor cell extracts [42], [43], it remained unclear whether it could be applied to extracts of complex multicellular organisms. Here we demonstrate the applicability of this screen to the identification of inhibitors of *Brugia* Hsp90. Cy3b-GA bound with similar kinetics to lysates prepared from adult *B. pahangi* and to extracts of tumor cells. Previous studies have shown that Hsp90 in normal cells possesses a very low affinity for inhibitors such as PU24FCl (10–50 fold less compared to tumor cell Hsp90) [44] or GA [22]. This precludes a direct comparison in the FP assay of *Brugia* Hsp90 with that found in normal tissues, but does suggest that Hsp90 inhibitors could have a selective effect on the parasite. Binding to *Brugia* Hsp90 was specific and could be inhibited by small molecules that bind in the N-terminal ATP pocket of Hsp90, thus validating the potential of the assay as a HTS for novel inhibitors of Hsp90. Several studies have now shown that Hsp90 from the free-living nematode *C. elegans* has a low affinity for GA in pull-down assays [14], [19], [31] and the assay was further validated by the inability of *C. elegans* extracts to bind cy3b-GA. The molecular basis of the differing sensitivities of nematode Hsp90s to N-terminal inhibitors has not been resolved but may reflect differences in post-translational modification of Hsp90, or interactions with different co-chaperones or client proteins [31].

Hsp90 is an attractive target in tumor cells for a number of reasons: because of the interaction of Hsp90 with a range of essential client proteins, inhibition of a single molecular species inhibits multiple pathways [45], which may reduce the likelihood of resistance developing. While nothing is currently known of Hsp90 clients in *Brugia*, yeast two-hybrid screens and analysis of genetic interactions have identified a number of likely Hsp90 targets in *C. elegans* (http://www.wormbase.org/db/gene/interaction?list=WBGene00000915), including transcription factors, nuclear hormone receptors and kinases. Efforts are currently on-going to identify Hsp90 clients in *Brugia*. Secondly, Hsp90 in tumor cells appears to have an increased affinity for drugs such as GA, a phenomenon which may relate to the conformation of Hsp90 in multi-chaperone complexes in tumor cells compared to normal cells, where it exists in a free uncomplexed state [22]. It is interesting to note that the affinity of *Brugia* Hsp90 for GA in this study was very similar to tumor cell Hsp90, but our studies indicated that other inhibitor classes, such as the purine-scaffold series, have different affinities for *Brugia* Hsp90 and human cellular Hsp90. This suggests that *Brugia* Hsp90 may be selectively targeted and supports HTS efforts towards the identification of novel chemotypes with enhanced potency and selectivity. The purine-scaffold derivatives used to elucidate the potential selectivity for the human and *Brugia* Hsp90 species have been extensively tested both *in vitro* and *in vivo*. These studies, together with relevant drug metabolism and pharmacokinetic analyses, demonstrate no liability with respect to chemical stability [34], [43], [44]. There are several other Hsp90 inhibitors in development [18], including molecules based on radicicol [46], which also targets the N'-terminal ATP pocket, or novobiocin [47], an inhibitor that binds at the C'-terminus of Hsp90, with a completely different mode of action to GA [48].

The concentration of drug required to kill adult *Brugia* (500 nM for GA, 2.5 µM for PU-H71) was significantly higher than the IC_50_ calculated from the FP assay. In part, this may reflect issues related to the uptake of drug by adult worms. At least *in vitro*, it is probable that small molecules are taken up across the cuticle [49], while *in vivo* the intestine is functional [50]. Little is known of the factors that influence the permeability of the filarial cuticle to small molecules. In *Ascaris*, the lipophilicity of a compound or the degree of methylation of peptides are important determinants of transcuticular uptake in an in vitro system [51]. However, the rapid onset of GA-induced effects in adult worms (Mf output being affected within 24 h, [14]) indicates the efficient absorption of drug.

In conclusion we have demonstrated that a HTS developed for tumor cell Hsp90 is applicable to an important parasitic species, *Brugia*. Thus far the assay has been validated using a small number of compounds that compete with labelled GA for binding to the *Brugia* Hsp90 N-terminal ATP binding pocket. Hsp90s are generally highly conserved and nematodes are no exception [31]. Hsp90 from *B. malayi* is 99.7% identical to *B. pahangi* Hsp90, validating the use of the model species in a screen for compounds that will target the human parasite. Given the format of the assay, the ability to utilise whole worm extracts and the relatively modest cost, the FP assay could now be applied to the identification of novel inhibitors of *Brugia* Hsp90 or adapted for other parasites where Hsp90 is a possible target, such as *Plasmodium falciparum* [25] and other important pathogens [52].
